# Supplementary material for: Light phase detection with on-chip petahertz electronic networks
Source: Nat Commun. 2020 Jul 8;11:3407. doi: 10.1038/s41467-020-17250-0 (PMC7343884; doi:10.1038/s41467-020-17250-0)
Supplement: Supplementary file 1 — Supplementary Information [file 41467_2020_17250_MOESM1_ESM.pdf]

Supplementary Information: Light Phase Detection with On-Chip Petahertz Electronic Networks

Yujia Yang[1], Marco Turchetti[1], Praful Vasireddy[1], William P. Putnam[1,2,3], Oliver Karnbach[1], Alberto Nardi[1], Franz X. Kärtner[3,4], Karl K. Berggren[1], Phillip D. Keathley[1]*

[1]Research Laboratory of Electronics, Massachusetts Institute of Technology, Cambridge, MA, USA
[2]Department of Electrical and Computer Engineering, University of California, Davis, Davis, CA, USA
[3]Department of Physics and Center for Ultrafast Imaging, University of Hamburg, Hamburg, Germany
[4]Center for Free-Electron Laser Science and Deutsches Elektronen-Synchrotron (DESY), Hamburg, Germany
*email: pdkeat2@mit.edu

Supplementary Note 1: Determination of the bow-tie nano-gap size

The bow-tie nano-gap sizes were measured from SEM images. To determine the gap size, we first performed a line scan across the bow-tie nano-gap (Supplementary Fig. 1a). From the grayscale value along the line, the nano-gap size was determined as the FWHM gap size (Supplementary Fig. 1b).

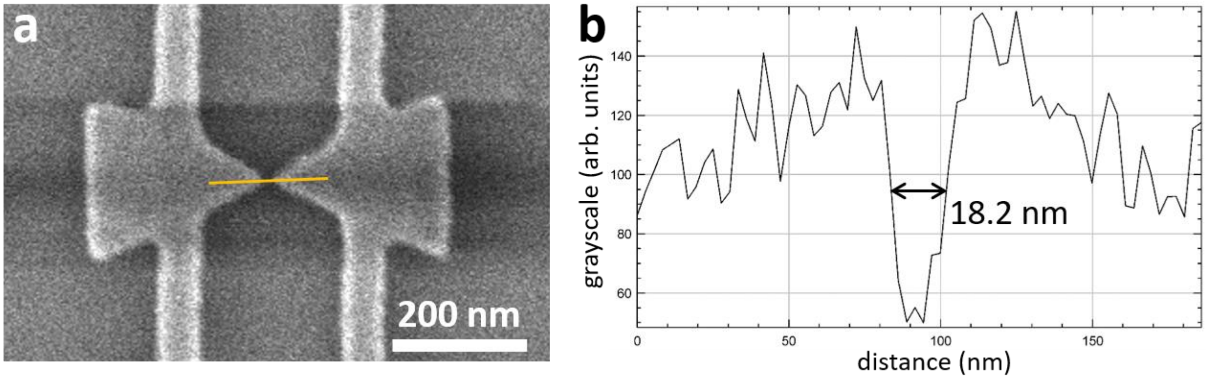

Supplementary Figure 1. Determination of the bow-tie nano-gap size. **a**, SEM image of a fabricated bow-tie nanoantenna. **b**, Grayscale value along a line scan (yellow line in **a**) across the nano-gap. The FWHM gap size is 18.2 nm.

Supplementary Note 2: Further details of optical simulation of nanoantennas

Optical simulation of the nanoantenna arrays in Fig. 1 (main text) used dimensions taken from the SEM images of a fabricated sample. The nanotriangle altitude was 245 nm, the base was 190 nm, and the bow-tie nano-gap was 50 nm. The sharp corners of the nanotriangle were rounded to avoid singularities and to better imitate fabricated samples.

Supplementary Fig. 2 shows the simulated extinction spectra, besides the field-enhancement spectra shown in Fig. 1e (main text), of the plasmonic bow-tie nanoantenna arrays with different connecting wire positions. When the connecting wires are added, the extinction spectra (Supplementary Fig. 2) splits into two peaks, corresponding to the bow-tie and wire modes. The spectral separation of the two peaks is small and they merge into a single peak, if the connecting wire position is near the center of the nanotriangle (e.g. X_{wire} = 120 nm). The spectral separation of the two peaks increases, with the bow-tie mode being blue-shifted and the wire mode being red-shifted, when the connecting wire position is close to the nanotriangle tip (e.g. X_{wire} = 50 nm) or nanotriangle base (e.g. X_{wire} = 200 nm). As expected from the discussions in the main text, placing the connecting wire close to the center of the nanotriangle leads to minimal perturbation of the bow-tie plasmonic mode. There is also a slight shift between the extinction peaks with respect to the field-enhancement peaks. For photoemission from nanoantenna devices, we tuned the field-enhancement peaks so that they were close to the central wavelength of the excitation laser.

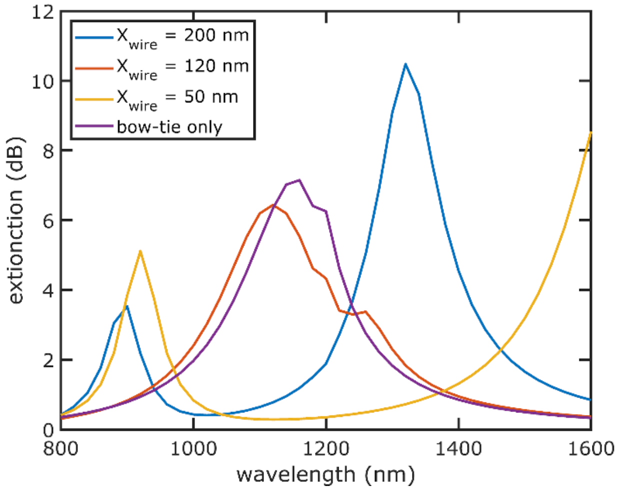

Supplementary Figure 2. Simulated extinction spectra. The extinction spectra for plasmonic bow-tie nanoantenna arrays with different connecting wire positions (X_{wire} labeled in Fig. 1b (main text)) are shown.

It has been shown that the plasmonic response can lead to a CEP shift compared to the free-space, incident optical pulse[1]. As the wire position strongly affects the plasmonic response, it is expected that the wire position should also affect the absolute CEP of the plasmonically enhanced waveform. This effect is confirmed by the simulation results shown in Supplementary Fig. 3. For an incident pulse with its CEP set to 0, the plasmonically enhanced waveforms have CEP shifts depending on the nanoantenna plasmonic response. The simulated absolute CEP values are -0.36π rad (-64.8 degrees),

-0.14π rad (-25.2 degrees), -1.33π rad (-239.4 degrees), and -0.35π rad (-63 degrees) for X_{wire} = 200 nm, 120 nm, 50 nm, and bow-tie without the wire, respectively. Our simulated CEP shift is in agreement with the results shown in Ref.[1].

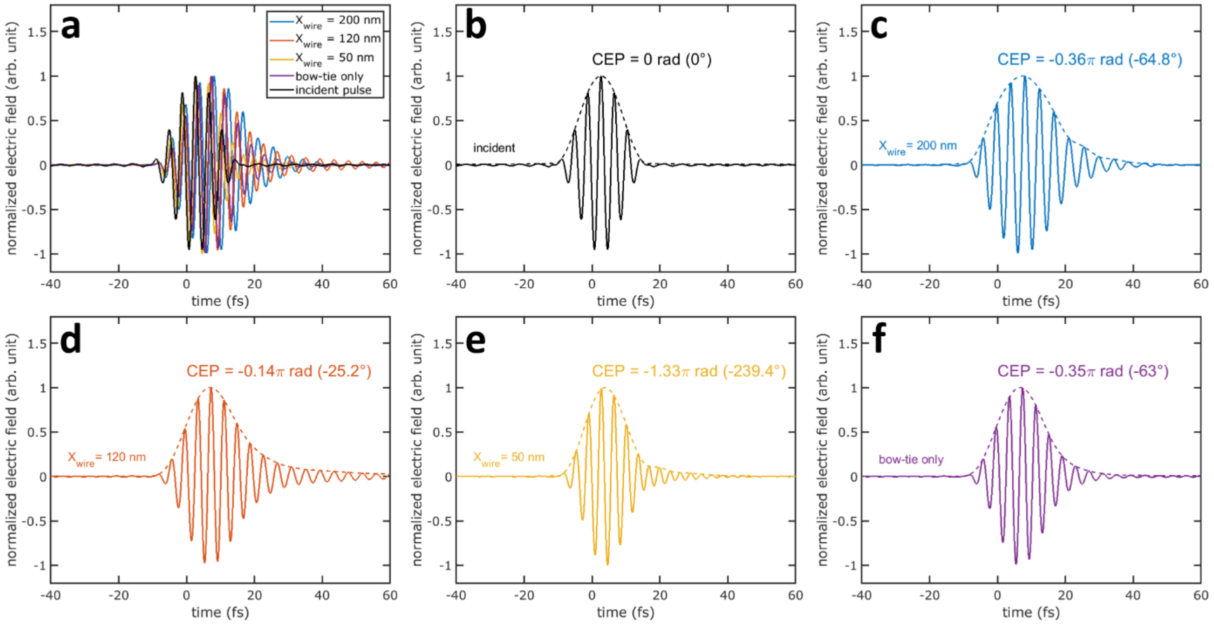

Supplementary Figure 3. Simulated optical and plasmonic waveforms. The electric field waveforms at the tip of the plasmonic bow-tie nanoantenna arrays with different connecting wire positions (X_{wire} labeled in Fig. 1b (main text)) are shown. **a**, Normalized waveforms for bow-tie nanoantennas with X_{wire} = 200 nm, 120 nm, and 50 nm, together with the waveform for bow-tie nanoantenna without the wire and the incident waveform. **b-f**, Each of the waveforms shown in **a**. The absolute CEP of each waveform is also labeled.

In the optical simulation with a parametric sweep of the connecting wire position (Fig. 1e-f (main text), Supplementary Fig. 2), the thin Ti adhesion layer was neglected to reduce the computation time. It has been demonstrated that a Ti adhesion layer could cause damping of the plasmonic resonance and reduce photoemission current from metallic nanostructures[2]. However, the spectral position of the plasmonic resonance is less affected by the adhesion layer. We investigated the effect of the Ti adhesion layer with optical simulations of electrically-connected bow-tie nanoantenna arrays (Supplementary Fig. 4). In agreement with previous reports, the plasmonic resonance intensity decreases when a Ti adhesion layer is present, while the spectral position of the resonance is less affected. Therefore, optical simulation of nanoantennas without an adhesion layer is sufficient to study the effect of the connecting wire position.

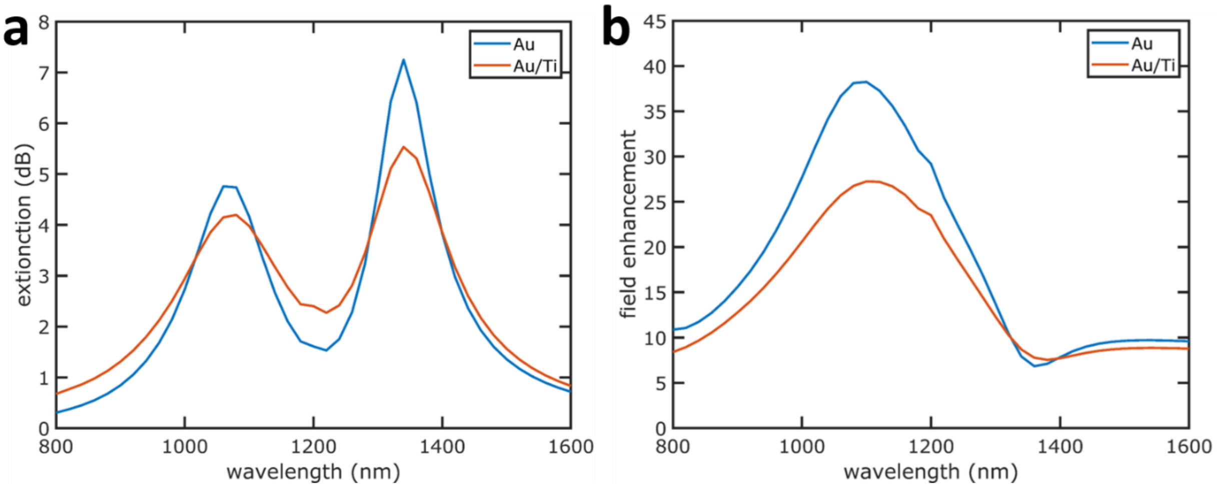

Supplementary Figure 4. Simulated plasmonic resonances with and without a Ti adhesion layer. **a**, Extinction spectra, and **b**, field-enhancement spectra of the nanoantenna with (Au/Ti) and without (Au) a 2-nm-thick Ti adhesion layer.

Supplementary Note 3: Electromigration of electrically shorted nanoantennas

We performed electromigration on the nanoantenna arrays shorted by connected bow-ties. The electromigration results largely depended on the number of connected bow-ties within the array. For an array with most of the bow-ties connected, all columns were shorted and the electromigration broke all connecting wires, leading to a complete open-circuit array (Fig. 2b (main text) and Supplementary Fig. 5a). For an array with just a few connected bow-ties and shorted columns, electromigration removed the shorted columns and kept the open-circuit columns, enabling the CEP-sensitivity of the array to be measured (Fig. 2c&d (main text)). For the connecting wires broken by electromigration, the break usually occurred at a position close to the contact pad (Fig. 2b-d (main text) and Supplementary Fig. 5a), while occasionally the break occurred at a position between the bow-ties (Supplementary Fig. 5b).

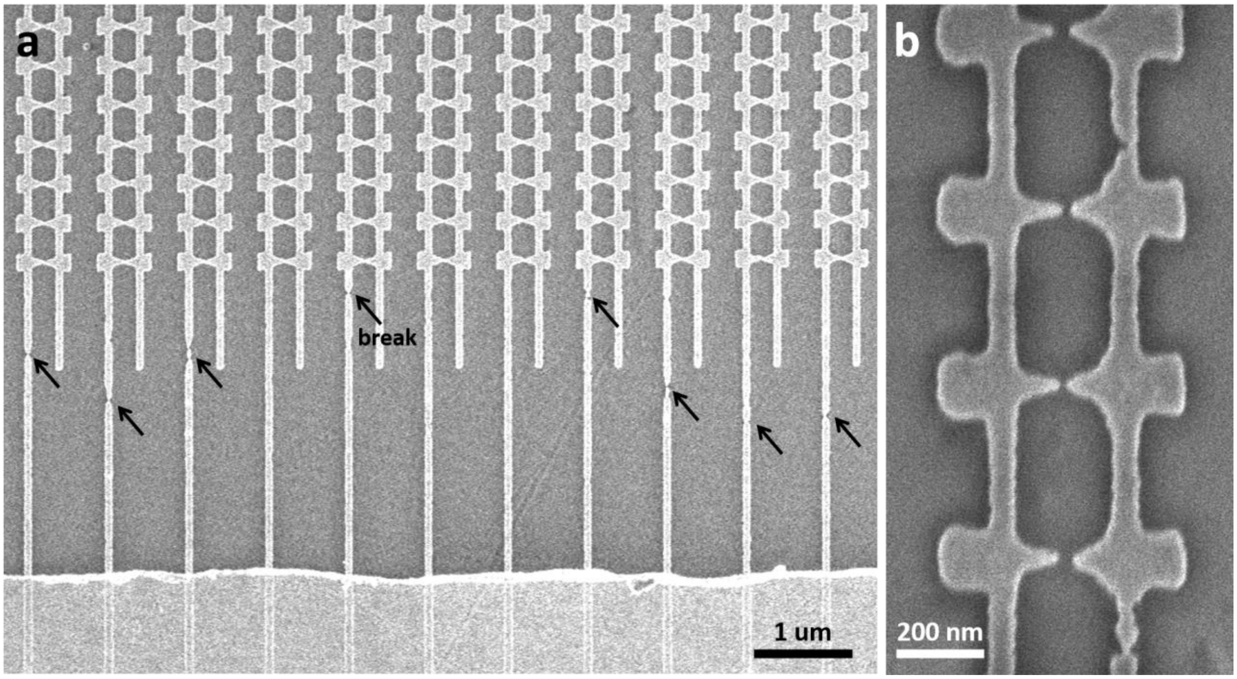

Supplementary Figure 5. Electromigration of electrically connected nanoantenna arrays. **a**, SEM image of a connected plasmonic nanoantenna array after electromigration. The electrical connecting wires were broken and disconnected during electromigration. Some of the breaks are indicated by black arrows. **b**, SEM image of a connecting wire broken by electromigration with the breaks between the bow-tie nanostructures.

Supplementary Note 4: Experimental setup

Supplementary Fig. 6 shows a schematic diagram of the experimental setup.

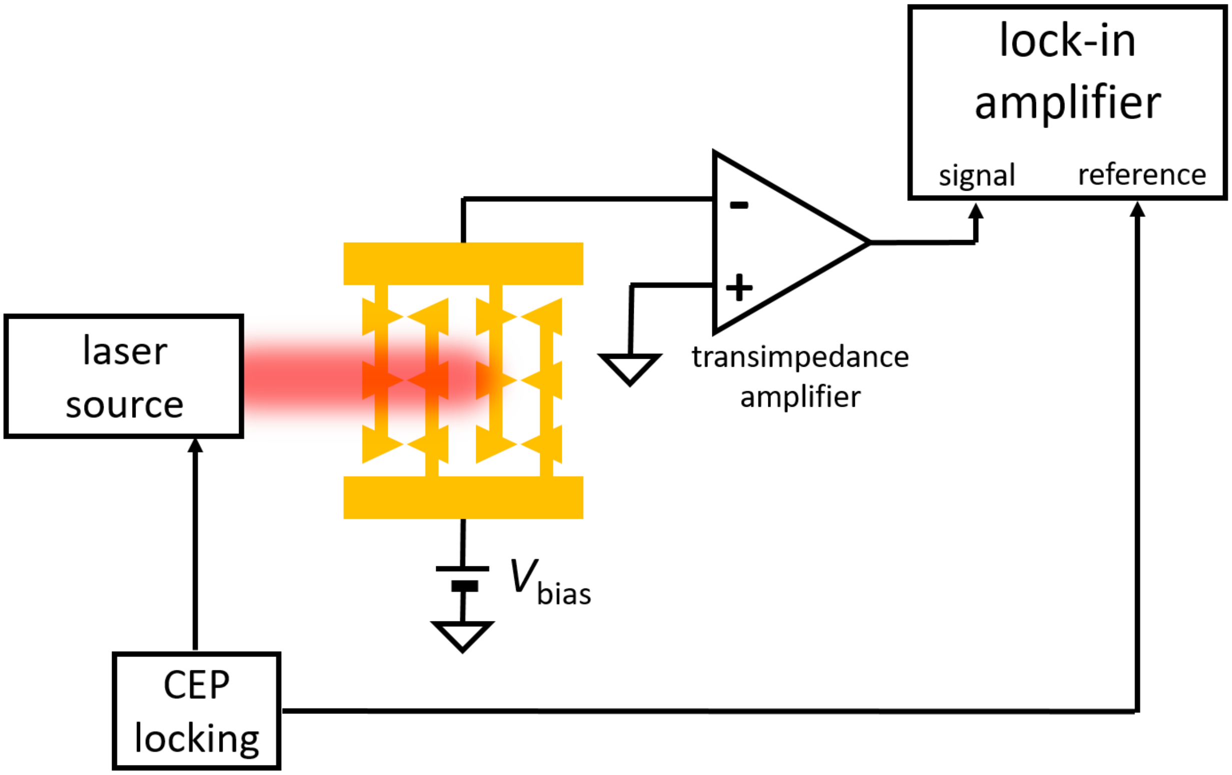

Supplementary Figure 6. Schematic diagram of the experimental setup. The photocurrent generated by the nanoantenna device was first amplified by a transimpedance amplifier, and then detected by a lock-in amplifier using the carrier-envelope-offset frequency as the reference frequency.

Supplementary Note 5: Ensuring adequate intensity for optical-field emission

To achieve CEP-sensitive emission, peak intensities are required such that the Keldysh parameter[3] $\gamma = \sqrt{\phi/2U_\mathrm{p}} < 1$, where ϕ is the emitter material work function (~5.1 eV for Au) and $U_\mathrm{p} = \mathrm{e}^2F^2\lambda^2/16\pi^2\mathrm{c}^2\mathrm{m}$ is the ponderomotive potential of the optical field at the emitter tip surface, where e is the electron charge, F the peak optical-field strength, λ the central wavelength of the optical pulse, c the speed of light, and m the electron mass. Even after obtaining a field-enhancement between 20-30 × for our plasmonic bow-tie antennas, the energy limitations of our current source require tight focusing to achieve $\gamma < 1$, meaning that we are not able to characterize CEP-sensitive photoemission while illuminating the entire emitter array.

Supplementary Note 6: Scaling CEP-sensitive signal and SNR with larger arrays and higher-energy pulses

Regarding the scaling of SNR of the CEP-sensitive current, we find that as long as f_{ceo} is high enough to be in the shot-noise limited regime due to the total emitted current, which scales linearly with I_{cep}, the SNR should scale linearly with the signal, and thus the array size, and inversely with the resolution bandwidth. Using a 3 Hz resolution bandwidth, we measured SNR values ranging from 20-30 dB when referenced to the shot-noise floor (see for instance the results in Supplementary Fig. 12). A 50×50 μm^2 array is roughly 350× larger than the current beam spot, meaning that by illuminating an entire 50×50 μm^2 we could maintain a 20-30 dB SNR over a resolution bandwidth of approximately 1050 Hz. To maintain the same peak intensity, this would require a pulse energy of ~70 nJ.

To achieve single-shot CEP tagging, sufficient CEP-sensitive electron charge yield per pulse is required to beat the shot-noise limit. Assuming Q_{emitted} is the total *emitted* charge yield per device per pulse and N_{d} is the number of nanoantenna devices being illuminated, the shot-noise charge per pulse is $\sqrt{Q_{\mathrm{emitted}} N_{\mathrm{d}}}$. We further define the CEP sensitivity k as the ratio of the CEP-sensitive charge yield per device per pulse to the total emitted charge per device per pulse, *i.e.* $k = Q_{\mathrm{cep}}/Q_{\mathrm{emitted}}$. As a result, the signal charge per pulse is $N_{\mathrm{d}}Q_{\mathrm{cep}}$ or $N_{\mathrm{d}}kQ_{\mathrm{emitted}}$. Hence, the SNR, measured as a power ratio, is $k^2 N_{\mathrm{d}} Q_{\mathrm{emitted}}$.

Assuming the peak signal measured in our experiment, we find that $Q_{\mathrm{cep}} = 0.1\ \mathrm{e}^-$, however after operating the devices for some time and scanning over the detector area, this peak signal degrades and we find an average $Q_{\mathrm{cep}} = 0.01\ \mathrm{e}^-$. The nanoantenna devices could operate stably at the degraded signal level for hours without further degradation. Using this degraded signal level, and again assuming a 70 nJ pulse illuminating an array of size 50×50 μm^2 (N_{d} = 7875), we plot the single-shot SNR *vs.* the CEP sensitivity k in Supplementary Fig. 7a. For comparison, we also plot the single-shot SNR assuming a 70 μJ pulse energy, comparable to the pulse energy typically used in single-shot CEP tagging experiments, and an even larger array size (1.5×1.5 mm^2). In our experiment, the estimated CEP sensitivity is roughly 10^{-5} to 10^{-4}. It can be seen that, with the larger array size and a typical pulse energy for CEP tagging, our device has a sufficient SNR for single-shot CEP measurement, and could potentially replace the bulky and expensive vacuum apparatus and electron time-of-flight spectrometers. For a smaller array size and low-pulse-energy (nJ) operation, the SNR needs to be improved by increasing the device array density, using the peak CEP-sensitive signal, and increasing the device CEP sensitivity.

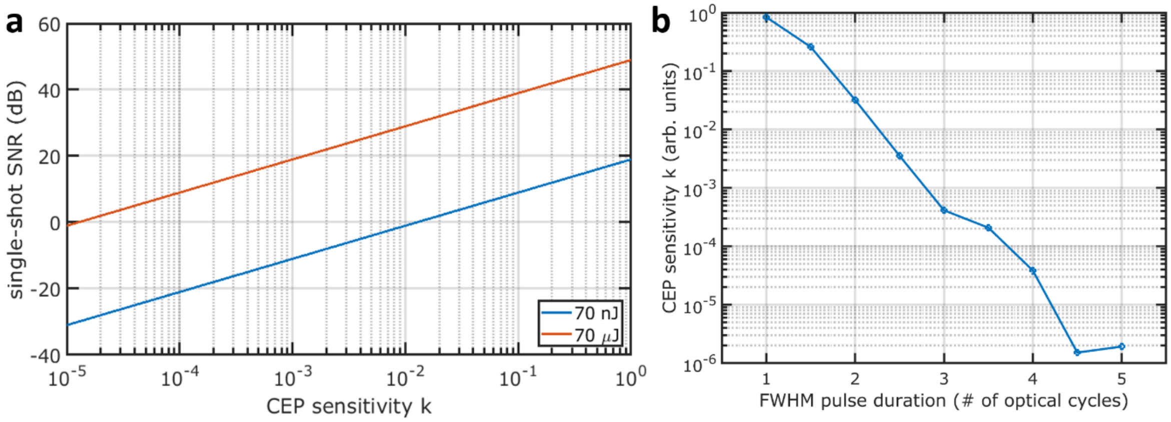

Supplementary Figure 7. Calculated device SNR and CEP sensitivity. **a**, CEP-sensitivity-dependent SNR of the nanoantenna device for single-shot CEP tagging. Two pulse energies, 70 nJ and 70 μJ, are considered. **b**, CEP sensitivity for various optical pulse durations. The optical cycle corresponds to a central wavelength of 1177 nm.

The CEP sensitivity *k* depends strongly on the optical pulse duration, as illustrated in Supplementary Fig. 7b. We calculated the CEP sensitivity based on a harmonic analysis of the Fowler-Nordheim photoemission current[4] from gold assuming transform-limited pulses of varying duration. While varying the pulse duration, the peak field was fixed at 42 GV/m assuming a 30× field enhancement and an incident field strength of 1.4 GV/m. It can be seen that a shorter optical pulse duration could significantly improve the CEP sensitivity and hence the device SNR. With sub-two-cycle optical pulses similar to those used in Refs.[5–7], we expect an improvement of one-to-two orders of magnitude in the CEP sensitivity and device SNR. For example, our calculation predicts a CEP sensitivity of ~0.1 for sub-two-cycle pulses. This sensitivity, together with a 50×50 μm^2 device array and a 70 nJ pulse energy, could lead to a SNR of ~10 dB for single-shot CEP tagging. Further investigations on the device CEP sensitivity and its dependence on the optical pulse duration and plasmonic resonance could shed light upon the future development of the nanoantenna devices capable of single-shot CEP tagging with a minute pulse energy.

Supplementary Note 7: Potential avenues for further improving device SNR

We note that for the room-temperature devices we studied in this work with gaps much larger than the deBroglie wavelength of the ground state electrons inside the nanoantennas, the emitted electron current from each triangle in the bow-tie pair was not strictly correlated, meaning that the ultimate noise floor was set by the shot-noise arising from the total emitted current from each triangle. Given the low CEP-sensitivity expected for the pulses used in this work (on the order of 10^{-4} to 10^{-5}), the total emitted current is significantly larger than the CEP-sensitive current. However, if the pulse at the tip surface could be sufficiently shortened, it would be possible to significantly enhance the CEP sensitivity and thus improve the observed SNR.

Another interesting avenue might be to pursue very short gaps within superconducting antennas such that a single electron state extending across the nanoantenna gap contributes to the tunneling current. In such a case, the emission from each triangle in the bow-tie gap, and thus its resultant noise, would be correlated, thus drastically reducing the shot-noise due to the total emitted current.

Supplementary Note 8: CEP-sensitive signals from more test devices

We measured the CEP-sensitivity of several samples from multiple fabrication batches, and obtained similar CEP-sensitive responses. Supplementary Fig. 8a&b show the phase and magnitude of I_{cep} from a nanoantenna device (Array 3) as a function of time with a laser pulse energy of 175 pJ. Discrete phase shifts were observed while translating the BaF_2 wedge every 20 s. The measured CEP-sensitive current magnitude had a peak value above 5 pA, and it degraded to ∼2 pA. Supplementary Fig. 8c&d show the phase and magnitude of I_{cep} while scanning the laser beam over Array 3. Similar to Fig. 3 (main text), the phase response of the array was relatively uniform, while there were hot and cold spots of the current magnitude. As a comparison, Supplementary Fig. 8e&f show the phase and magnitude of I_{cep} from another nanoantenna device (Array 4) with a larger nano-gap size. The measured CEP-sensitive current magnitude was ∼0.1 pA, and the phase response was noisier. A larger nano-gap led to a lower field-enhancement, and hence a lower CEP-sensitive photocurrent. However, the CEP-sensitive signal showed less degradation, possibly due to a reduced laser-reshaping effect resulting from a lower plasmonic enhancement.

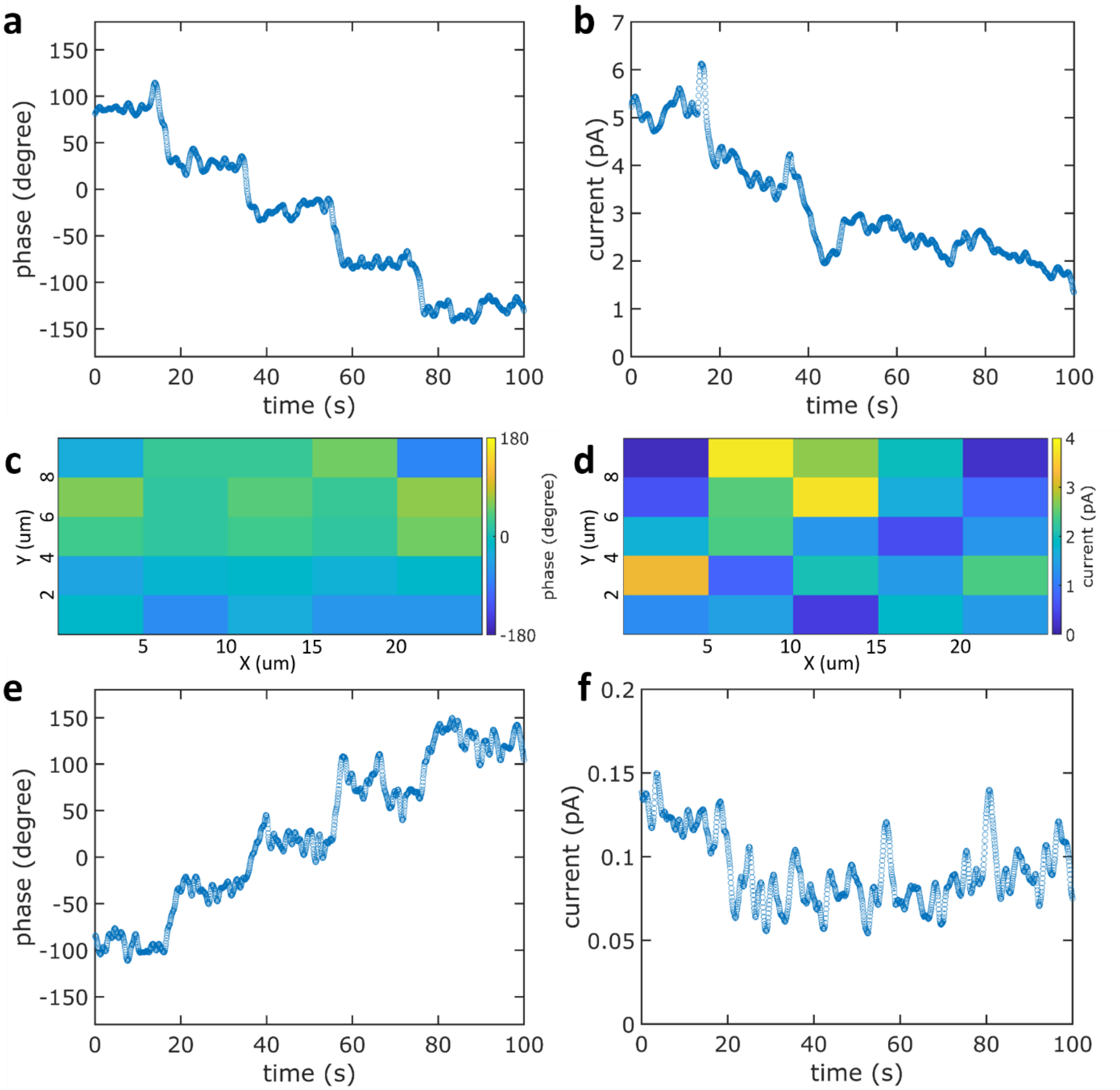

Supplementary Figure 8. CEP-sensitive photocurrent and nano-gap size. The CEP-sensitive currents I_{cep} from nanoantenna devices with various nano-gap sizes are shown. **a**&**b**, the phase and magnitude of I_{cep} from a nanoantenna device (Array 3) as a function of time with a laser pulse energy of 175 pJ. **c**&**d**, the phase and magnitude of I_{cep} while scanning the beam over Array 3. **e**&**f**, the phase and magnitude of I_{cep} from another nanoantenna device (Array 4) as a function of time with a laser pulse energy of 155 pJ. For all measurement results, the BaF_2 wedge was stepwise inserted or retracted every 20 s.

Supplementary Fig. 9 shows the phase and magnitude of I_{cep} from a nanoantenna device (Array 3) with various laser pulse energies. For a laser pulse energy of 175 pJ, the CEP-sensitive photocurrent had a peak value of ~5 pA and a stabilized value of ~2 pA, while for a laser pulse energy of 139 pJ or 123 pJ, the CEP-sensitive photocurrent was around 1 pA. Notably the CEP-sensitive signal showed

little or no sign of degradation at a low pulse energy, which suggests the lower pulse energies didn't reshape the nanoantennas.

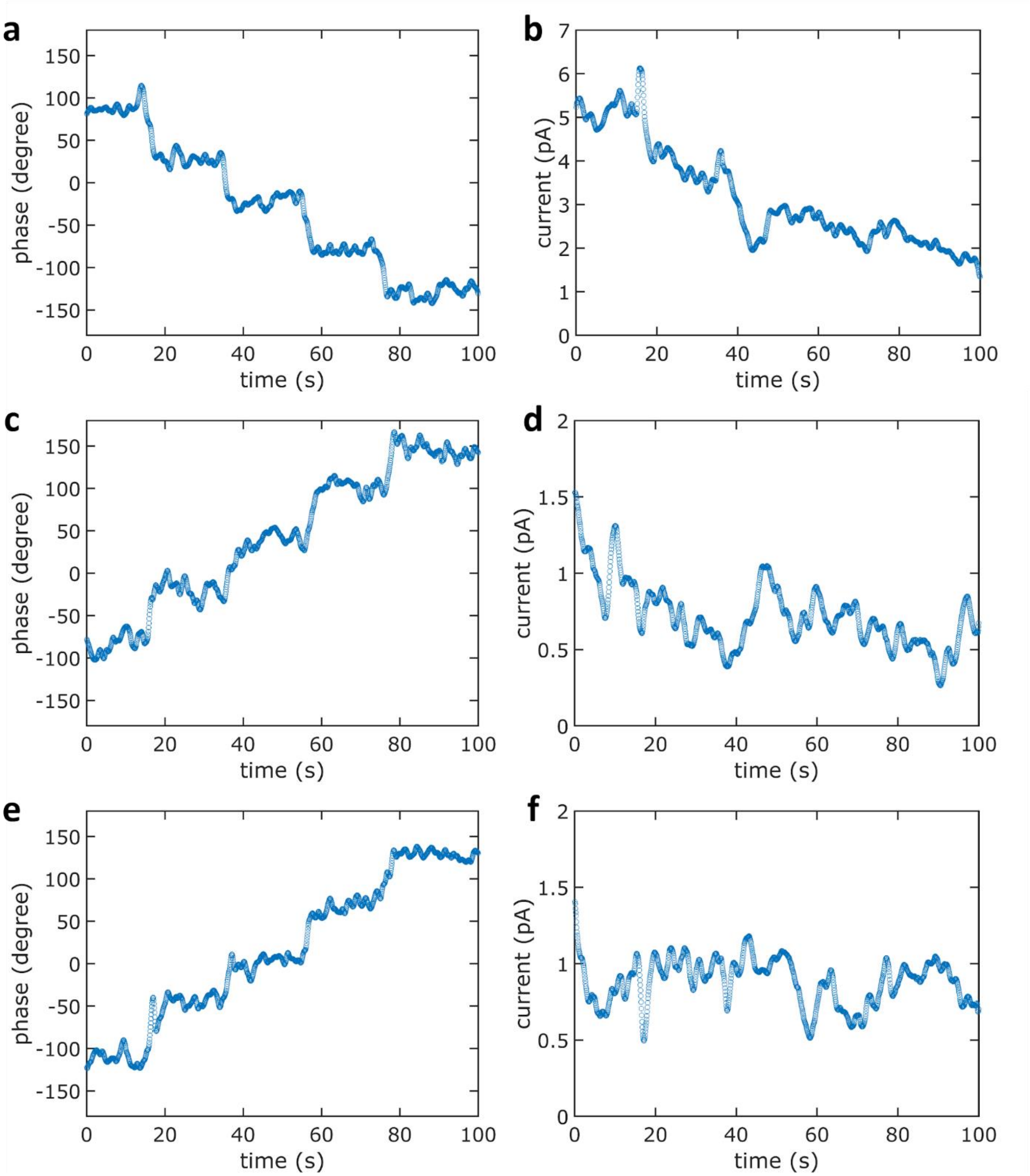

Supplementary Figure 9. CEP-sensitive photocurrent and laser pulse energy. The CEP-sensitive currents I_{cep} from nanoantenna devices under the illumination of laser pulses with various pulse energies are shown. **a**&**b**, the phase and magnitude of I_{cep} from a nanoantenna device (Array 3) as a function of time with a laser pulse energy of 175 pJ (the same as Supplementary Fig. 8a&b). **c**-**f**, the phase and magnitude of I_{cep} from the same nanoantenna device (Array 3) as a function of time with a laser pulse energy of 139 pJ (**c**&**d**) or 123 pJ (**e**&**f**). For all measurement results, the BaF_2 wedge was stepwise inserted or retracted every 20 s.

Uniformity of the CEP-response across an entire array is important for the scaling and multiplexing of the device networks. We raster-scanned the laser beam spot across the device arrays while collecting the CEP-sensitive photocurrent amplitude $|I_{\text{cep}}|$ and phase $\angle I_{\text{cep}}$. Supplementary Table 1 shows the mean values and standard deviations of the photocurrent amplitude, as well as the standard deviations of the photocurrent phase, for multiple device arrays. The low phase variations across each array indicate the scalability of our device with large-scale integration.

Device Array #	mean $\|I_{\text{cep}}\|$ (pA)	standard deviation of $\|I_{\text{cep}}\|$ (pA)	standard deviation of $\angle I_{\text{cep}}$ (mrad)
5	5.14	2.91	233
6	3.60	1.58	417
7	0.175	0.057	277
8	2.45	1.10	308
9	2.30	1.08	485
10	2.55	1.27	400
11	1.83	0.61	434

Supplementary Table 1. Uniformity of the device array. Measured mean values and standard deviations of the CEP-sensitive photocurrent amplitude, as well as the standard deviations of the photocurrent phase, for multiple device arrays are shown.

Supplementary Note 9: Laser-reshaping of nanoantennas and change of CEP-sensitivity

For most of the devices tested, we observed an increase of the bow-tie nano-gap after laser illumination. Supplementary Table 2 shows the gap sizes of 3 representative samples as fabricated and after laser illumination. The laser exposure dose was roughly 10^8 pulses with 78 MHz repetition rate and up to ~190 pJ pulse energy. Regardless of the as-fabricated gap sizes, the gap sizes after laser illumination always ended up in the 50-60 nm range. These similar gap sizes after laser illumination suggest the laser-induced reshaping was self-stabilized as the gap size increased and plasmonic enhancement decreased during laser illumination. Further investigations with more test samples and accurate laser exposure dose calibration are required to confirm this phenomenon.

Sample #	Gap size as fabricated (nm)	Gap size after laser illumination (nm)
1	45.0 ± 2.8	49.6 ± 7.1
2	50.5 ± 3.2	62.2 ± 11.9
3	39.3 ± 3.3	61.7 ± 5.7

Supplementary Table 2. Laser-reshaping of nanoantennas. Nano-gap sizes for 3 representative bow-tie nanoantenna arrays before and after laser illumination are shown.

We observed a degraded CEP-sensitivity of the nanoantenna devices during laser illumination. This degradation could be intuitively explained as the increased bow-tie gap size leading to a decreased field-enhancement, and hence a decreased photoelectron emission current. However, it has been recently reported that the CEP-sensitivity does not monotonically change with the optical field[4]. Supplementary Fig. 10 shows the simulated CEP-sensitive photocurrent magnitude $|I_{\mathrm{cep}}|$ with a varying peak incident optical-field strength for the nanoantenna array in Fig.4 (main text) before (blue) and after (orange) the photoemission measurement (similar to Fig. 4c (main text) but with a larger range of the incident optical field). For an incident optical field in the range of 13-40 GV/m, the nanoantenna device after illumination (with a larger nano-gap and a smaller field-enhancement) shows a higher CEP-sensitivity compared to the device before illumination, which has a vanishing CEP-sensitivity. Nevertheless, this counterintuitive behavior requires a large incident optical field (10× the optical field we used in our experiments). As we have already observed laser-induced reshaping and device degradation in the experiments, the counterintuitive, improved CEP-sensitivity after illumination is unlikely to occur in our devices.

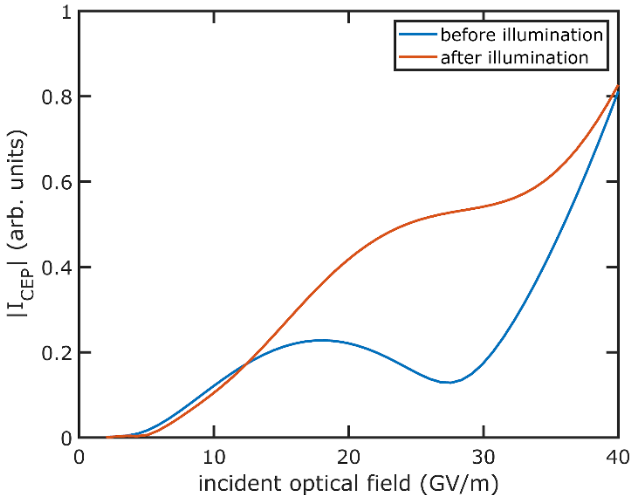

Supplementary Figure 10. Simulated CEP-sensitivity *versus* optical field strength. Simulated CEP-sensitive photocurrent magnitude $|I_{\mathrm{cep}}|$ *versus* the optical near-field for the nanoantenna array before and after photoemission measurement is shown. An incident optical field up to 40 GV/m is considered.

Supplementary Note 10: Photocurrent measurement with a DC bias scan

We measured the photocurrent response at 0 Hz corresponding to the total average current detected $I_{0,\mathrm{detected}}$ as a function of the DC bias voltage V_{bias} between the two nanotriangle emitters in the bow-tie pair (e.g. Fig. 5b (main text)). Supplementary Fig. 11 shows the measurement of the total average photocurrent with a DC bias scan for two device arrays. The V_{bias} value that gives $I_{0,\mathrm{detected}} \approx 0$ A varies slightly across the samples.

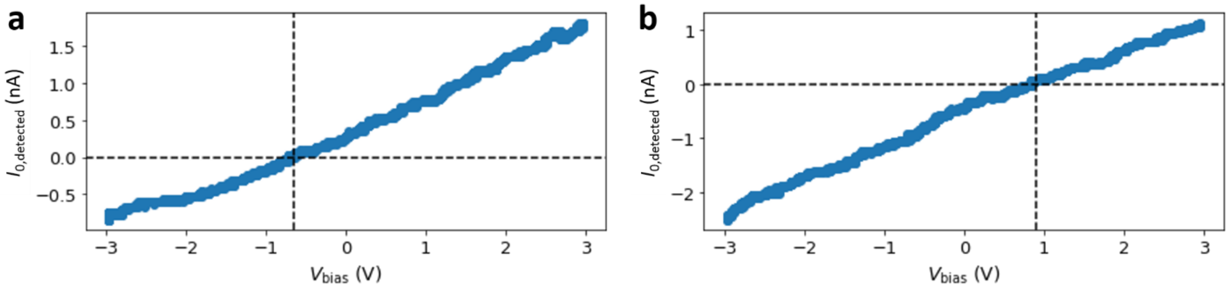

Supplementary Figure 11. Photocurrent with a DC bias. Plots of $I_{0,\mathrm{detected}}$ demonstrate the use of a DC bias voltage V_{bias} to null the total average detected photocurrent signal. The DC bias scans for two arrays are shown (**a**, **b**). The V_{bias} value that gives $I_{0,\mathrm{detected}} \approx 0$ A varies slightly across the two arrays.

Supplementary Note 11: Further investigations of the noise floor

In the manuscript we argue that the noise arises from shot-noise resulting from $I_{0,\text{emitted}}$. To provide further evidence of this, we recorded current spectra showing the CEP beat note (here at 120 Hz) and surrounding noise floor when exposed to ~190 pJ pulses with various bias conditions as shown in Supplementary Fig. 12. Note that the biased case is for V_{bias} = 3 V, the unbiased case for V_{bias} = 0 V, the unlocked case is for no bias and no CEP locking, and the noise floor was recorded with no optical beam directed onto the devices. We note that the resolution bandwidth for these measurements was 3 Hz. The noise floor was found to be on the order of just 30-40 fA, agreeing with measurements of the noise-floor using the lock-in amplifier, and around one order of magnitude reduced from the noise floor when illuminated. This confirms that our observed noise indeed arises from the devices under illumination.

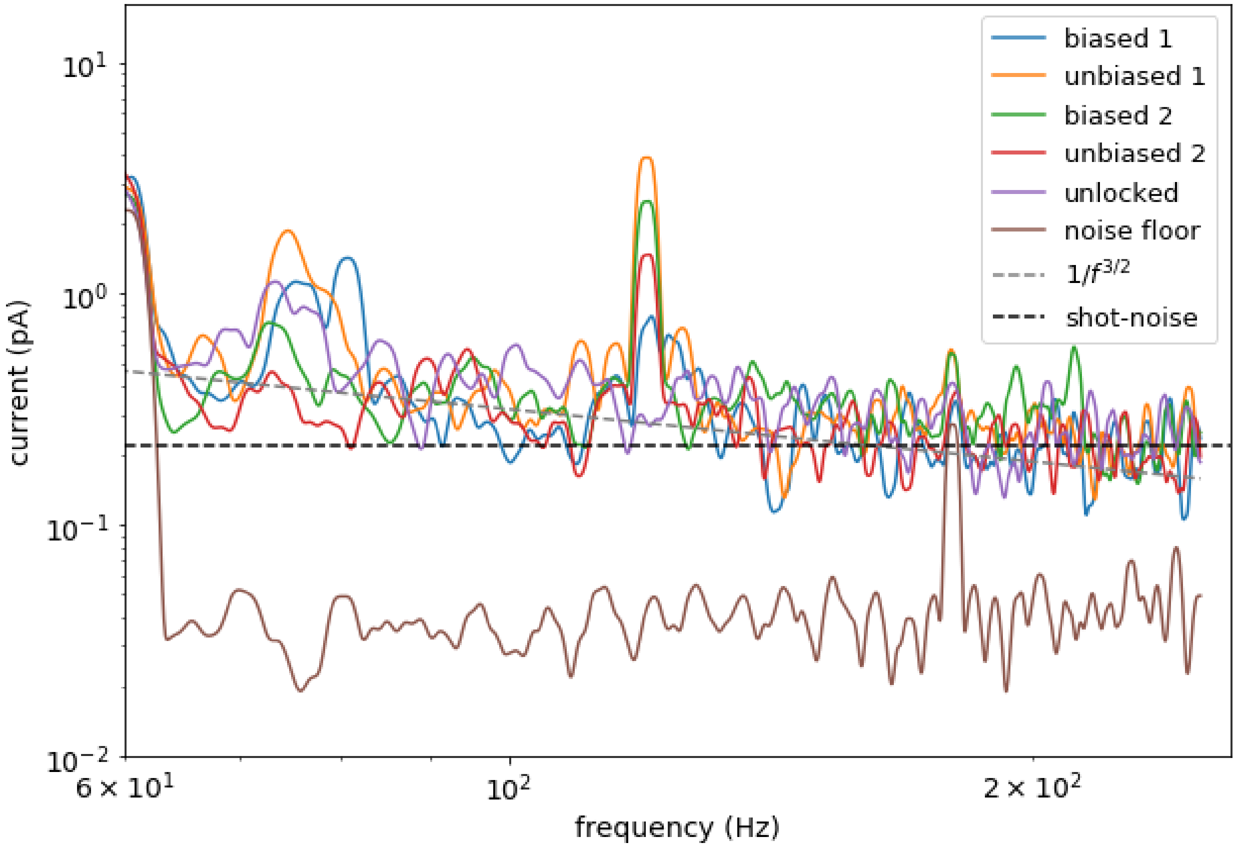

Supplementary Figure 12. Photocurrent and noise spectra. The current spectra show the CEP beat note (at 120 Hz here) and the background noise floor for various settings of V_{bias}. The biased curves are for V_{bias} = 3 V and the unbiased curves for V_{bias} = 0 V. Two sets of measurements were performed (biased & unbiased 1, biased & unbiased 2), and the device degradation caused a reduction of the signal peak. The unlocked case is unbiased and was taken with the CEP unlocked. The noise floor was taken with the beam blocked (*i.e.* no illumination of the devices by the laser beam). Reference curves for $1/f^{3/2}$ (dashed gray) and shot-noise (dashed black) scaling are shown indicating a transition near 150 Hz for this particular sample.

As usual, there was a slow degradation in CEP, but we found that there was no correlation between the strength of the CEP note and the bias in general up to V_{bias} = 3 V. Importantly, the measurements clearly show no correlation between the noise floor and the bias despite an observed increase in $I_{0,detected}$ by several orders of magnitude when the bias is on as opposed to when it is off (see Fig. 5 (main text)). This indicates that the noise arises either from the illumination or the total emitted current $I_{0,emitted}$, which would be unaltered by a mild bias.

Furthermore, we can compare the CEP response and relative noise floor to the single-triangle devices reported in Ref.[8]. In Supplementary Fig. 13, we compare single-sided triangular devices to the electrically-connected devices used in this work. The resonance of the single-sided triangles was similar to the electrically-connected devices (near 1158 nm). We purposefully chose a region of electrically-connected bow-tie emitters having a similar CEP-sensitive photocurrent under full illumination to the single-sided triangle devices. As shown in Supplementary Fig. 13, the SNR and noise floor are almost identical. This highlights that the dominant noise source is not common-mode in origin (for example noise due to energy fluctuations of the optical source) as common-mode noise sources should have been reduced significantly using the symmetric electrically connected devices.

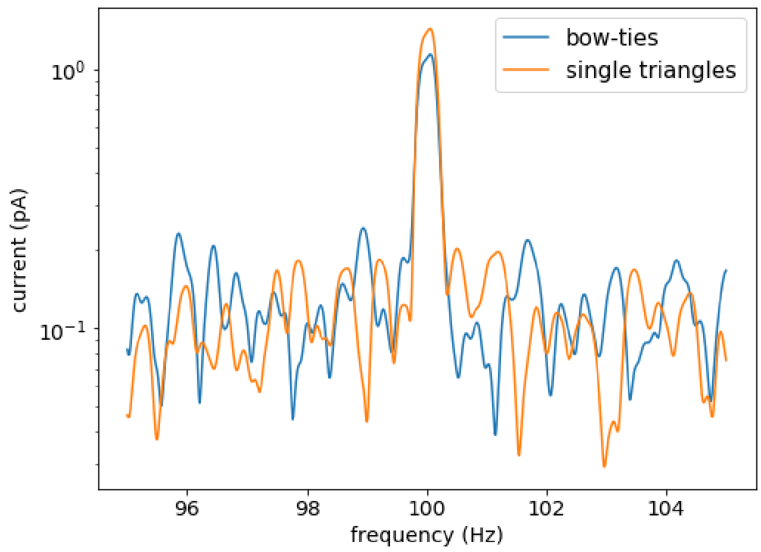

Supplementary Figure 13. CEP-sensitive photocurrents from bow-tie and triangular nanoantennas. This plot compares single triangle emitters (see Ref.[8]) to the electrically connected bow-tie devices. A region of devices was chosen such that the CEP-sensitive photocurrent is similar between the two cases. They were both illuminated with the same optical conditions (peak energy near 200 pJ). Both devices exhibit nearly identical noise floor under similar conditions, emphasizing that the dominant noise source is not common-mode in origin. A resolution bandwidth of 0.3 Hz was used in the measurement.

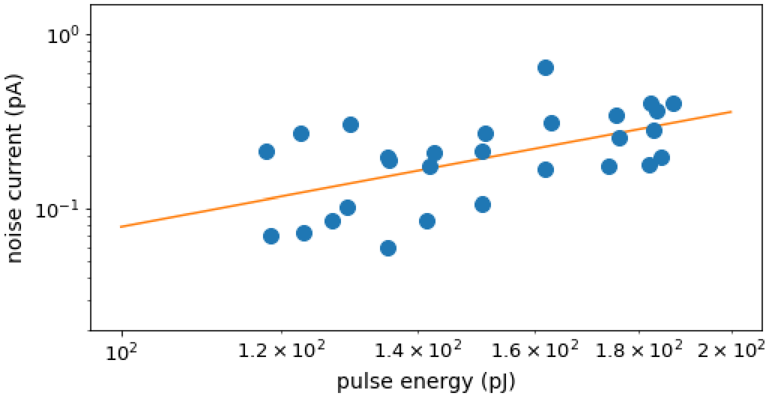

Supplementary Figure 14. Noise current and laser pulse energy. This plot shows the noise current as a function of pulse energy incident on the nanostructures. The trend-line fit indicates a scaling proportional to approximately $P^{2.18}$. However, for intensity-induced thermal noise, one would expect a scaling with $P^{0.5}$.

Finally, the noise-floor level is examined as a function of the incident pulse energy in Supplementary Fig. 14. The data shown is in fact the same as that from Array 1 in Fig. 5 (main text), only now plotted as a function of pulse energy. There are two key reasons we rule out thermal noise from absorption of the incident laser power as the primary contributor to the noise floor. First, there is visually less correlation between P (the incident pulse energy) and I_{noise} as between $I_{0,\text{detected}}$ and I_{noise}. Second, when fitting the scaling factor, we find that the noise grows proportionally to $P^{2.18}$ while one would expect laser-induced thermal noise to grow as the square-root of the temperature, and thus as $P^{0.5}$ [9].

References

1. Ludwig, M. *et al.* Sub-femtosecond electron transport in a nanoscale gap. *Nat. Phys.* **16**, 341–345 (2020).
2. Hobbs, R. G. *et al.* High-Yield, Ultrafast, Surface Plasmon-Enhanced, Au Nanorod Optical Field Electron Emitter Arrays. *ACS Nano* **8**, 11474–11482 (2014).
3. Keldysh, L. Ionization in the field of a strong electromagnetic wave. *Sov Phys JETP* **20**, 1307–1314 (1965).
4. Keathley, P. D. *et al.* Vanishing carrier-envelope-phase-sensitive response in optical-field photoemission from plasmonic nanoantennas. *Nat. Phys.* 1–6 (2019) doi:10.1038/s41567-019-0613-6.
5. Paasch-Colberg, T. *et al.* Solid-state light-phase detector. *Nat. Photonics* **8**, 214–218 (2014).
6. Rybka, T. *et al.* Sub-cycle optical phase control of nanotunnelling in the single-electron regime. *Nat. Photonics* **10**, 667–670 (2016).
7. Higuchi, T., Heide, C., Ullmann, K., Weber, H. B. & Hommelhoff, P. Light-field-driven currents in graphene. *Nature* **550**, 224–228 (2017).
8. Putnam, W. P., Hobbs, R. G., Keathley, P. D., Berggren, K. K. & Kärtner, F. X. Optical-field-controlled photoemission from plasmonic nanoparticles. *Nat. Phys.* **13**, 335–339 (2017).
9. Van Der Ziel, A. Flicker Noise in Electronic Devices. in *Advances in Electronics and Electron Physics* (eds. Marton, L. & Marton, C.) vol. 49 225–297 (Academic Press, 1979).
